# Predicting black soldier fly larvae biomass and methionine accumulation using a kinetic model for batch cultivation and improving system performance using semi-batch cultivation

**DOI:** 10.1007/s00449-021-02663-y

**Published:** 2021-12-04

**Authors:** Lydia Palma Miner, Jesus Fernandez-Bayo, Ferisca Putri, Deb Niemeier, Heather Bischel, Jean S. VanderGheynst

**Affiliations:** 1 Department of Biological and Agricultural Engineering, University of California, One Shields Ave., Davis, CA 95616 USA; 2 Department of Civil and Environmental Engineering, University of California, Davis, CA USA; 3 Department of Civil and Environmental Engineering, University of Maryland, College Park, MD USA; 4 Department of Bioengineering, University of Massachusetts, Dartmouth, MA USA

**Keywords:** Insect cultivation, Kinetic model, Almond byproduct, Methionine

## Abstract

Global demand for poultry and associated feed are projected to double over the next 30 years. Insect meal is a sustainable alternative to traditional feeds when produced on low-value high-volume agricultural byproducts. Black soldier fly (BSF) larvae (*Hermetia illucens* L.) are high in protein and contain methionine, an essential amino acid that is critical to poultry health. BSF larvae can be grown on many organic residues, however, larvae growth and quality vary based on feedstock and cultivation processes. Experiments were completed to monitor temporal changes in BSF larvae growth and composition using almond hulls as a growth substrate under batch and semi-batch processes and with varying substrate carbon to nitrogen ratio (C/N). A logistic kinetic growth model was developed to predict larval biomass and methionine accumulations during batch production. Estimated ranges of model parameters for larvae maximum specific growth rate and carrying capacity were 0.017–0.021 h^−1^ and 9.7–10.7 g larvae kg^−1^ hulls dry weight, respectively. Methionine content in larvae increased from 11.1 to 17.1 g kg^−1^ dry weight over a 30-day batch incubation period. Larvae-specific growth and yield increased by 168% and 268%, respectively, when cultivated in a semi-batch compared to a batch process. Increasing C/N ratio from 26 to 40 increased density of methionine content in larvae per unit feedstock by 25%. The findings demonstrate a logistic model can predict larvae biomass accumulation, harvest time can achieve specific methionine contents, and a semi-batch process is more favorable for larvae biomass accumulation compared to a batch process.

**Supplementary Information:**

The online version contains supplementary material available at 10.1007/s00449-021-02663-y.

## Introduction

The human global population is projected to reach 9.5 billion by 2050 which will put increasing demands on food production [1]. Finding methods that increase food production on existing natural resources is critical for addressing growing needs for nutritious food. Producing insect biomass requires less land and resources compared to the production of other protein sources. For example, the land required to produce 1 kg of beef, soy, mealworm and black soldier fly (BSF) larvae is 12.5, 3.5, 0.60 and 0.22 square meters, respectively [2–5]. BSF are desirable for insect farming compared to other insects, because they have a relatively fast life cycle, they are not a vector for disease, they exhibit “self-harvesting” behavior, and they have a favorable composition to meet nutritional requirements of humans and animals [6, 7]. BSF larvae are high in protein, fat, amino acids, essential fatty acids and minerals [8, 9]. Many studies have demonstrated BSF larvae meal is comparable to soy and fishmeal and can be substituted into diets for poultry, fish and humans [10–12]. BSF larvae can also grow on a variety of organic substrates and low-value, high-volume agricultural residues, and could play a role in closing the loop between organic waste recycling and food production.

One potential insect growth substrate that is abundant in California is almond hulls, which are derived from the processing of almonds. The almond kernel is surrounded by a shell and an outer hull. In 2018, the California almond industry produced approximately 1 million metric tons of almond kernels and over 2 million metric tons of almond hulls [13]. While almond hulls are a suitable feedstock for BSF larvae cultivation, larvae growth and composition vary with growth conditions [14, 15]. For example, larvae harvest dry weight increased from 5.4 to 9.2 mg larvae^−1^ when carbon to nitrogen (C/N) ratio of almond hull feedstock was decreased from 49 to 16 by amending hulls with urea and incubation at 28 °C [15]. Also, methionine content in harvested larvae increased from 4.15 to 4.63 g kg^−1^ dry weight when temperature was decreased from 32 to 28 °C and C/N was adjusted to 16 [15]. It is important to understand the impacts of cultivation environment on larvae biomass accumulation and composition to enable the consistent production of BSFL biomass for animal feeds and other products [7].

Kinetic models are one way to predict BSF larvae biomass accumulation in a system. For example, a study confirmed the logistic function could be used to compute growth rates for BSF larvae by validating data sets from a variety of experiments including variables of temperature, feed quality, feeding rate, feed moisture content and airflow rate [16]. The Verhulst logistic growth model was used to find the specific growth rates of BSF larvae grown on mixtures of degassed sludge, industrial organic waste and commercial chicken feed to estimate costs of growth, maintenance metabolism, rates of feed assimilation and net growth efficiency [17]. Another study monitored the daily growth of BSF larvae reared on ten different substrates and found the Richards model was the best fit growth model and that larvae followed an S-shaped growth curve [18]. The logistic growth model has been used to describe BSF larvae dry weight over time from day 0 after inoculation until reaching prepupae and maximal weight [17, 19]. Furthermore, prior observations of BSF larvae growth as a function of time indicate growth follows an exponential growth pattern and reaches a stationary phase during pre-pupation [20]. These findings suggest a logistic model could be used to predict BSF larvae growth on almond hulls for improving system performance for large-scale production. Furthermore, accounting for nutrient accumulation in a prediction model would significantly benefit animal feeding industries.

The main objectives of this research were to (1) develop a kinetic model for BSF larvae growth to facilitate the prediction of larvae biomass and methionine accumulation using almond hulls as substrate, (2) measure the dynamic changes in nutrient content in BSF larvae to provide insight into how harvest time could be managed to overcome varying nutrient content in larvae, (3) and test the impact of batch (single substrate feeding) versus semi-batch (multiple substrate feedings) cultivation on larvae and methionine production. The results demonstrate that BSF larvae and methionine accumulation follow a logistic model under specific environmental conditions, altering harvest time is one way to achieve a specific methionine content in larvae, and producing larvae in a semi-batch process enhances insect biomass and methionine production compared to batch production.

## Materials and methods

### Acquisition and processing of almond hull feedstock

A Monterey variety of almond hulls and shells was obtained from a processor in Buttonwillow, CA from the 2017 harvest and used as larvae cultivation feedstock. The hulls were ground using a hammer mill with a 6.35 mm screen and then stored in airtight plastic bags. The as-received moisture content of the material was 18.2% (dry basis). Composition analyses were done by UC Davis Analytical Laboratories (Davis, CA). Total nitrogen and carbon were measured according to AOAC 972.43, through the flash combustion method using thermal conductivity/IR detection (LECO FP-528 and TruSpec CN Analyzers) [21]. Total crude protein was calculated from the nitrogen content with a protein factor of 6.25 [22]. Fat was measured using the Randall modification of the standard Soxhlet extraction [23]. Calcium was measured using nitric acid/hydrogen peroxide microwave digestion and determination by Inductively Coupled Plasma Atomic Emission Spectrometry (ICP-AES) [24, 25]. Total sugar was measured by enzymatically hydrolyzing at 55 °C with amyloglucosidase for 12 h and analyzed by high performance liquid chromatography (HPLC) with mass selective detection [26]. Total starch was calculated from the total and free glucose [26]. Neutral detergent fiber was measured using Association of Official Agricultural Chemists (AOAC) 2002–04 [23]. The acid detergent fiber and acid detergent lignin were measured using AOAC 973.18 [27]. The material was reported to have 8.6 g kg^−1^ total nitrogen, 411.1 g kg^−1^ total carbon, 53.8 g kg^−1^ protein, 20.8 g kg^−1^ fat, 2.8 g kg^−1^ calcium, 100.0 g kg^−1^ total sugar, 5.50 g kg^−1^ total starch, 348.5 g kg^−1^ acid detergent fiber, 409.0 g kg^−1^ neutral detergent fiber, and 67.8 g kg^−1^ acid detergent lignin.

### BSF larvae rearing

Larvae rearing methods were conducted as described in detail elsewhere using eggs supplied by Trevor Fowles (Department of Entomology, University of California, Davis, USA) [14]. Larvae were reared on chicken feed (Purina Premium Poultry Feed Layena Crumbles, Purina Animal Nutrition LLC, Shoreview, MN) at a moisture content of 500 g kg^−1^ wet basis and incubated at 28 °C. 7–9-day-old larvae were used in the experiments. In the first experiment, larvae were separated from feed using 1 and 2 mm sieves. To have consistent larvae size in the second experiment, larvae were manually separated from feed based on size. Prior to inoculation onto hulls, samples of larvae were collected for moisture content measurement and average dry weight per larvae.

### Feedstock preparation and incubations

Feedstock was amended with distilled water and nitrogen prior to incubation. Urea (Fisher Scientific Company LLC, Hampton, NH) was added to distilled water to achieve target carbon to nitrogen (C/N) ratios in the hull mixtures. Urea was used as a model nitrogen source in these studies rather than an organic waste source of nitrogen, because urea could be obtained consistently and distributed uniformly throughout the hulls. Adjusting C/N of hulls using urea was found to significantly impact larvae growth in prior studies [15]. From each mix, three random samples were collected to measure pH and moisture content prior to larvae inoculation. Table 1 lists the C/N ratios, moisture content, initial pH of hulls, inoculation density of larvae, average number of larvae per bioreactor, average initial larvae size, aeration rate and incubation temperature for each experiment. Larvae were cultivated in 1500 mL bioreactors as described in detail elsewhere [14, 15]. Humidified air was provided to the bioreactors by bubbling house air through distilled water and metered to each bioreactor with polycarbonate rotameters (5–50 mL min^−1^, Dwyer Instruments, Michigan City, IN). Clear poly tubing was secured to the inlet and joined with a 6.4 mm diameter black porous soaker drip line to ensure uniform aeration through the bed of feedstock.

**Table 1:** Parameters for experiments

Variables	Experiment 1: kinetic model	Experiment 2: C/N feeding
Carbon to nitrogen ratio	26	26, 33, 40, 47
Moisture content (g kg^−1^ wet basis)	690	680
Initial pH of hull mixture	4.72	4.79
Inoculation density [g larvae kg^−1^ hulls (dry weight)]	1.3	1.9^a^
Average number of larvae in inoculum (# larvae per bioreactor)	143	100
Average larvae weight in inoculum (mg dry weight/larvae)	1.8	3.8
Aeration rate (mL min^−1^ g^−1^ dry weight)	0.20	0.21
Incubation temperature (°C)	28	28

^a^Inoculation density based on the total dry weight of hulls added throughout the experiment

The first experiment was designed to measure larvae biomass and methionine accumulation as a function of time in batch culture. One bioreactor was harvested every 48 h for a total of 15 bioreactors (time points over 30 days). The feeding rate was calculated by dividing the initial hulls dry weight by the number of larvae and incubation period and was approximately 67 mg dry/larvae/day over a 30-day cultivation period and 100 mg dry/larvae/day over a 14-day cultivation period (the latter used in the model validation). Larvae biomass and methionine and cystine contents were measured at each time point. Data from this experiment were used to develop the kinetic model and inform the hull feeding amount and the time of feeding for the second experiment.

The second experiment was designed to determine the impact of semi-batch cultivation and C/N ratio on larvae growth and composition. The C/N ratio of the initial feedstock fed to all bioreactors was 26. For the second feeding, which occurred at 150 h, the feedstock was adjusted to achieve four different C/N ratios (26, 33, 40 and 47) prior to feeding. The time of the second feeding was determined based on the logistic growth period observed in the first experiment. One batch control treatment was fed all the hulls during the initial feeding with no additional hulls added throughout the cultivation period. This batch treatment was prepared to validate the kinetic parameters found from the first experiment and to compare results from batch versus semi-batch cultivation. The total amount of hulls fed for all treatments in this experiment (200 dry grams) was split evenly between the two feedings for a feeding rate of approximately 143 mg dry/larvae/day. Three replicate bioreactors were prepared for each treatment for a total of 15 bioreactors. The responses included specific larvae growth, average larvae harvest weight, larvae yield, hull consumption, methionine and cystine contents of harvested larvae (g amino acid kg^−1^ larvae), methionine accumulation (g larvae methionine content kg^−1^ hulls), and total methionine produced. The cultivation studies ran for 14 days. Temperature inside each bioreactor was monitored using iButton temperature loggers (Thermochron iButton DS1921G-F5#, Embedded Data Systems, Lawrenceburg, KY) wrapped in thermoplastic wrap (Parafilm M Wrapping Film, Fischer Scientific, Hampton, NH). Temperature data were logged every 15 min (Fig. S1).

### Larvae harvest and analysis

At the end of the experiments, the contents of bioreactors were frozen at − 20 °C. Larvae were separated and counted at a later date; total larvae weight and numbers of larvae recovered per reactor were recorded. Larvae moisture content was measured for each treatment. Separated larvae were stored at − 20 °C and homogenized with an oscillating ball mill (MM400, Retsch Inc., Newtown, PA). The homogenized larvae were freeze dried (VirTis 50-SRC-5, SP Scientific, Warminster, PA) for 4 days. The methionine and cystine contents were measured at the UC Davis Genome Center, Molecular Structure Facility (Davis, CA, USA) using performic oxidation with hydrolysis [28].

### Spent substrate analysis

At the end of the experiments, samples of spent hull substrate were analyzed for moisture content and pH using methods as previously described [28]. For both experiments, moisture content and pH were analyzed at the time of larvae harvest. For the first experiment, samples of hulls were freeze dried and methionine and cystine were measured using the same method described above for larvae.

### Data analysis

Larvae biomass and methionine accumulation kinetics during batch production were modeled using the logistic equation (Eqs. 1 and 2):

1 $$\frac{\mathrm{d}{X}_{b}}{\mathrm{d}t}={{X}_{b} \mu }_{\mathrm{max},b}\left(1-\frac{{X}_{b}}{{X}_{\mathrm{max},b}}\right),$$

2 $${X}_{m}={f}_{m}{X}_{b},$$

3 $$\frac{\mathrm{d}{X}_{m}}{\mathrm{d}t}={{X}_{m} \mu }_{\mathrm{max},m}\left(1-\frac{{X}_{m}}{{X}_{\mathrm{max},m}}\right),$$

where X_b_ = larvae biomass density (g larvae kg^−1^ hulls (dry weight)), *f*_m_ = methionine content in larvae biomass (g larvae methionine g^−1^ larvae), *X*_m_ = methionine density (g larvae methionine kg^−1^ hulls (dry weight)), *t* = time (hr), *X*_max*,b*_ = maximum larvae biomass density (g larvae kg^−1^ hulls (dry weight)), *X*_max*,m*_ = maximum methionine density during batch growth (g larvae methionine kg^−1^ hulls (dry weight)), *μ*_max*,b*_ = maximum specific larvae biomass accumulation rate (h^−1^), and *μ*_max*,m*_ = maximum specific methionine accumulation rate (h^−1^). It was assumed that methionine content remained constant during the period of logistic growth. Larvae methionine accumulation differs from the larvae methionine content, such that the accumulation represents the total larvae methionine content per total feedstock, or the density of methionine content in the system. The logistic equations were integrated using an initial density of *X*_*b*_ = *X*_*0,b*_ and *X*_*m*_ = *X*_*0,m*_ at *t* = 0 to obtain the following forms (Eqs. 4 and 5):

4 $${X}_{b}=\frac{{X}_{\mathrm{max},b}{X}_{0,b}{\mathrm{e}}^{{\mu }_{\mathrm{max},b}t}}{{X}_{\mathrm{max},b}-{X}_{0,b}+{X}_{0,b}{\mathrm{e}}^{{\mu }_{max,b}t}},$$

5 $${X}_{m}=\frac{{X}_{\mathrm{max},m}{X}_{0,m}{\mathrm{e}}^{{\mu }_{\mathrm{max},m}t}}{{X}_{\mathrm{max},m}-{X}_{0,m}+{X}_{0,m}{\mathrm{e}}^{{\mu }_{\mathrm{max},m}t}}.$$

Parameter estimates in Eqs. (4) and (5) were determined using experimental data and the *nlinfit* nonlinear regression function in MATLAB (MATLAB R2017b, MathWorks, Natick, MA).

The larvae yield and hull consumption were calculated as described in detail elsewhere [14]. For the second experiment, responses for yield, hull consumption, average larvae harvest dry weight, final larvae composition, methionine accumulation and total methionine produced were analyzed as a function of C/N ratio. Interactions between C/N ratio and feeding frequency was also investigated. Standard least squares regression analyses and Tukey’s HSD tests were performed using JMP-IN software (version Pro 12, SAS, Cary, NC). The significance level was set to 0.05.

## Results

### Batch process

#### Kinetic model development

**Fig. 1:**
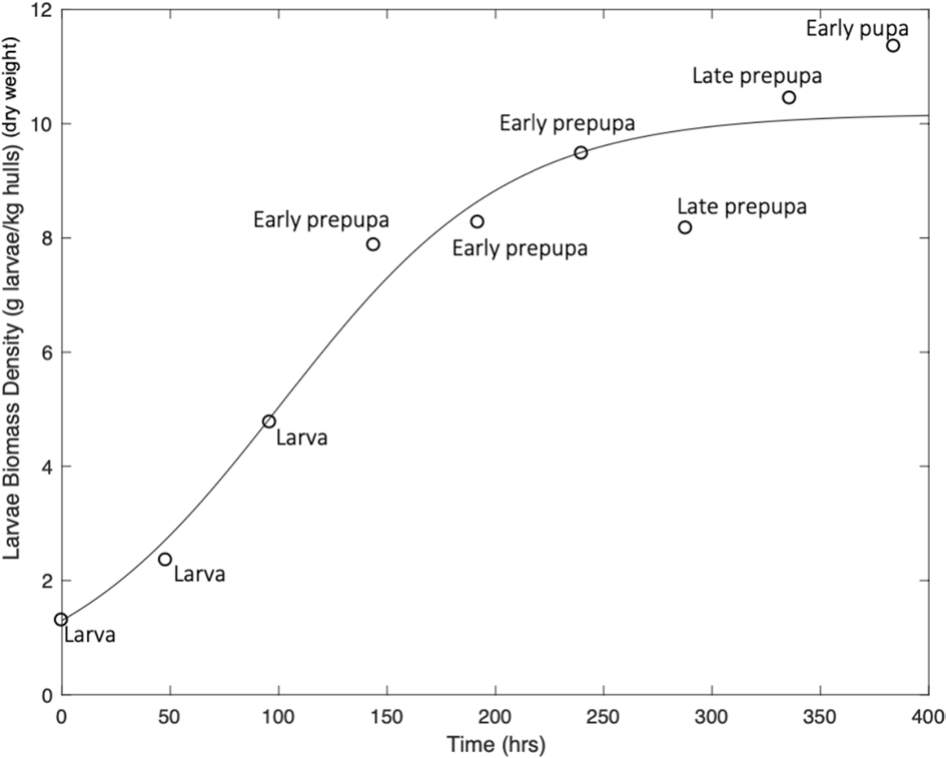
Larvae growth on almond hulls in a batch process in Experiment 1. The points represent measured larvae biomass density for a single bioreactor with the approximate life cycle stage determined through visual inspection. The line represents the model fit of the logistic equation (Eq. 4)

The logistic growth model fit larvae biomass accumulation data through the early pupa life stages for batch cultivation, shown in Fig. 1. The exponential growth phase was observed to be during the first 144 h of growth and the stationary phase to be between approximately 144–400 h of growth. Biomass data were used to estimate parameters in the logistic growth model (Table 2). The parameter estimates were 10.2 g larvae kg^−1^ hulls (dry weight) for ${X}_{\mathrm{max},b}$, and 0.019 h^−1^ for ${\mu }_{\mathrm{max},b}$. The root mean squared error was 4.4 g larvae kg^−1^ hulls indicating a meaningful fit between the model and the biological data.

**Table 2:** Estimated kinetic parameters for larvae biomass and methionine accumulation for larvae grown on almond hulls

Parameter	Units	Estimate ± SE
${\mu }_{\mathrm{max},b}$	h^−1^	0.019 ± 0.002
${X}_{\mathrm{max},b}$	g larvae kg^−1^ hulls (dry weight)	10.2 ± 0.5
${\mu }_{\mathrm{max},m}$	h^−1^	0.015 ± 0.002
${X}_{\mathrm{max},m}$	g larvae methionine kg^−1^ hulls (dry weight)	0.14 ± 0.01

**Fig. 2:**
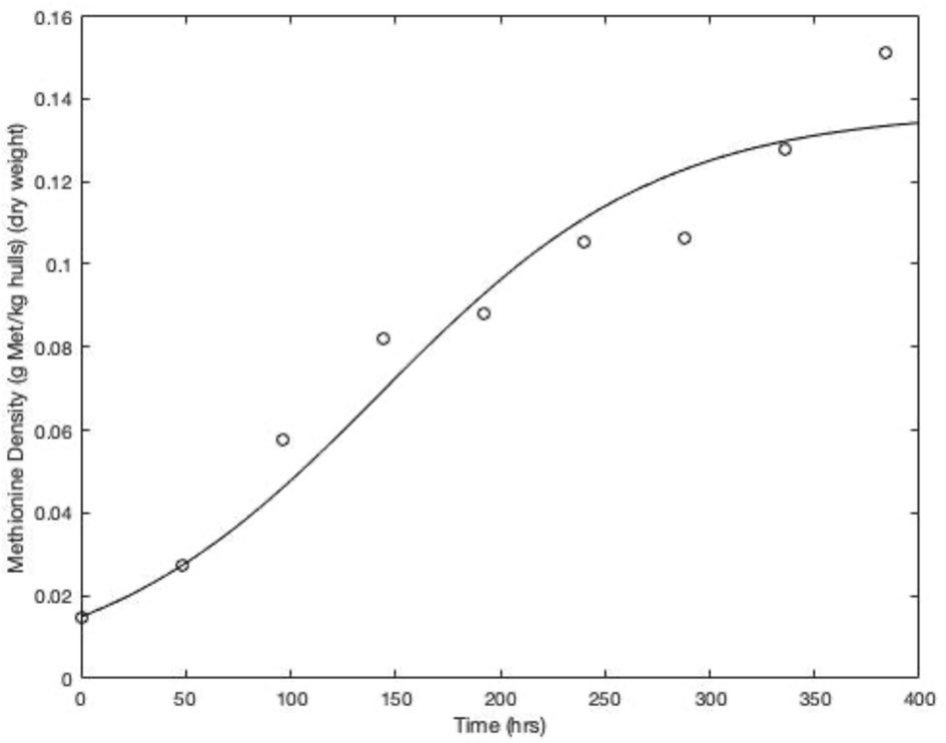
Methionine accumulation in harvested larvae biomass in Experiment 1. The points represent the measured methionine accumulation in harvested larvae for a single bioreactor and the line represents the model fit of the logistic equation (Eq. 5)

**Fig. 3:**
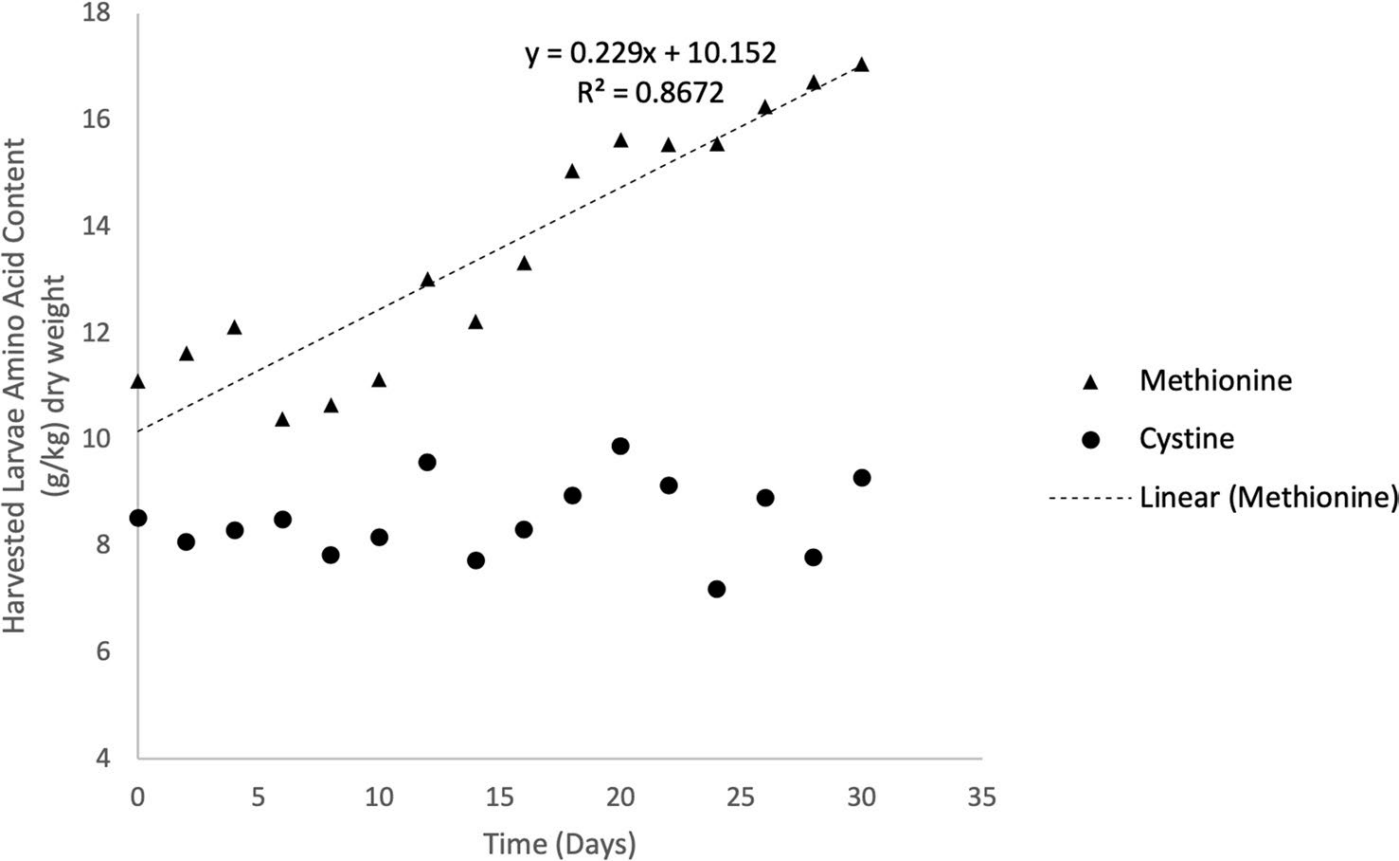
Dynamic changes in methionine and cystine contents in black soldier fly larvae grown on almond hulls in a batch process in Experiment 1. Total 30-day duration represents time after inoculation on hulls. Time zero represents start of experiment, where larvae are approximately 5–7 days old

Methionine accumulation data for batch cultivation of larvae are provided in Fig. 2. These data were used to estimate parameters in the logistic growth model (Table 2). The parameter estimates were 0.137 g larvae methionine kg^−1^ hulls (dry weight) for ${X}_{\mathrm{max},m}$ and 0.015 h^−1^ for ${\mu }_{\mathrm{max},m}$. The root mean squared error was 0.06 g larvae methionine kg^−1^ hulls indicating a meaningful fit between the model and the experimental data. The difference between ${\mu }_{\mathrm{max},b}$ and ${\mu }_{\mathrm{max},m}$ may have been due to the change in methionine content in larvae during batch growth (Fig. 3).

#### Validation of kinetic model

Batch cultivation data from the control treatment of the second experiment were used to validate the biomass accumulation model using Eq. (4). Estimates for *X*_max,*b*_ and *μ*_max,*b*_ (Table 2) and the initial condition for *X*_*0,b*_ for the second experiment (1.9 g larvae kg^−1^ hulls (dry weight)), predicted the larvae biomass density to be 10.1 g larvae kg^−1^ hulls (dry weight) after 336 h of growth. The measured larvae biomass density in the control treatment in the second experiment at that time was 9.3 g larvae kg^−1^ hulls (dry weight) with a standard deviation of 1.2 g larvae kg^−1^ hulls (dry weight). The percent error was approximately 7.9% between the expected and experimental values.

Batch cultivation data from the second experiment were also used to validate the methionine accumulation model. Using Eq. 5, estimates for *X*_max,*m*_ and *μ*_max,*m*_ (Table 2) and the initial condition for *X*_0,*m*_ for the second experiment [0.023 g larvae methionine kg^−1^ hulls (dry weight)], the predicted methionine density was 0.13 g larvae methionine kg^−1^ hulls (dry weight) after 336 h of growth. The measured methionine density in the control treatment of the second experiment was 0.10 g larvae methionine kg^−1^ hulls (dry weight) with a standard deviation of 0.018 g larvae methionine kg^−1^ hulls (dry weight). The percent error was approximately 23% between the expected and experimental values.

#### Changes in larvae composition over time

Methionine content in larvae ranged from 10.4 g kg^−1^ dry larvae to 17.1 g kg^−1^ dry larvae over the 30-day batch experiment (Fig. 3). Linear regression of larvae methionine content with respect to time showed a significant first-order relationship (Table 3, Fig. 3). Cystine content of harvested larvae averaged 8.5 g kg^−1^ dry larvae throughout the study (Fig. 3). There were no significant changes in harvested larvae cystine content with respect to time (*P* > 0.05).

**Table 3:** Linear regression of larvae methionine content, hull consumption, hull methionine content and hull cystine content and as a function of time (days)

Variable	Parameters	Values (standard error)	Time range for linear regression (days)	*P* value	*R*^2^
Hull consumption (% dry basis)	Slope, % days^−1^ intercept, %	0.055(0.003) − 0.037(0.012)	0–6	0.03	0.99
Larvae methionine content (g Met kg^−1^ larvae)	Slope, g kg^−1^ days^−1^ Intercept, g kg^−1^	0.23 (0.02) 10.2(0.4)	0–30	< 0.0001	0.87
Hull methionine content (g Met kg^−1^ hulls)	Slope, g kg^−1^ days^−1^ Intercept, g kg^−1^	0.02(0.01) 4.35(0.18)	0–30	0.046	0.29
Hull cystine content (g Cys kg^−1^ hulls)	Slope, g kg^−1^ days^−1^ Intercept, g kg^−1^	0.028(0.009) 3.81(0.16)	0–30	0.009	0.45

#### Hull consumption and composition over time

**Fig. 4:**
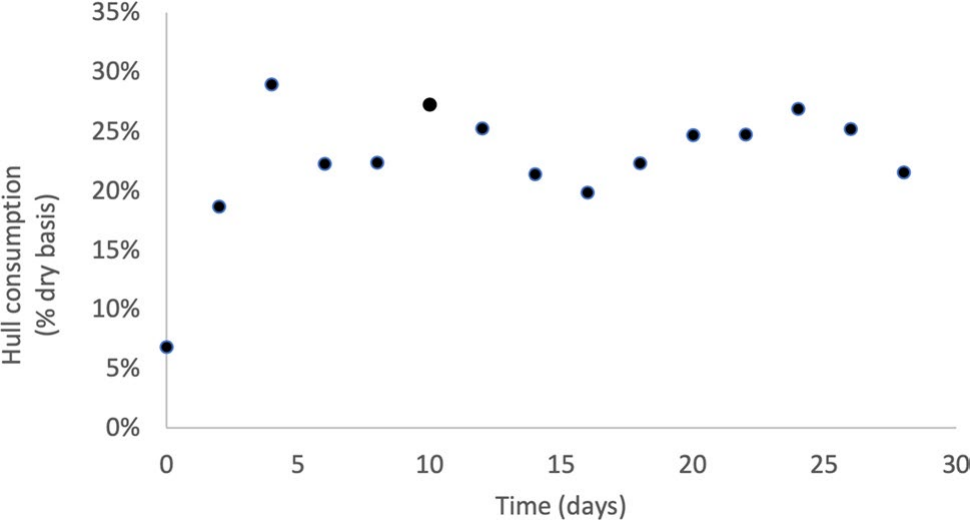
Consumption of almond hulls by larvae and microorganisms during 30-day batch cultivation in Experiment 1. Each point represents the total hull consumption associated with one bioreactor for the specific time period

**Fig. 5:**
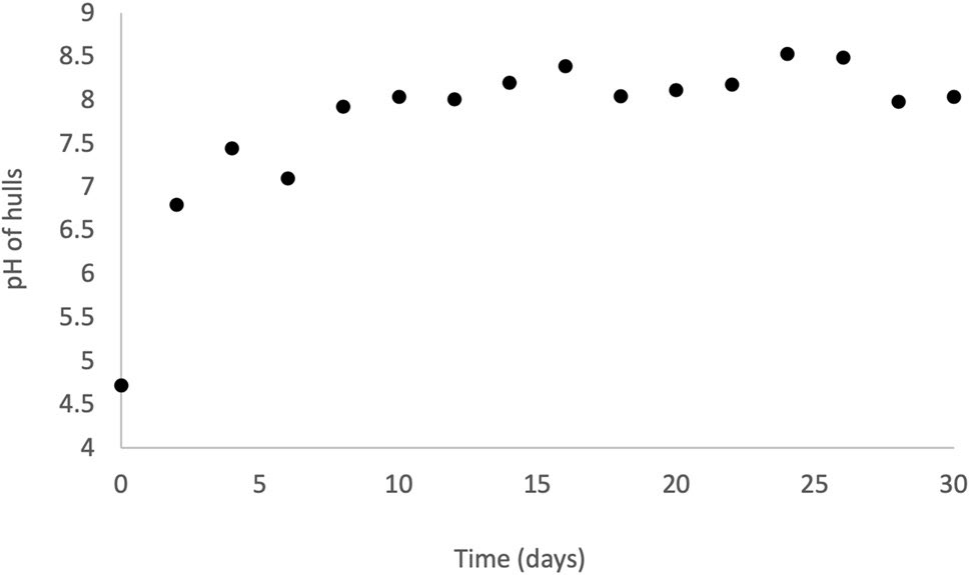
Change in hull pH during 30-day batch cultivation in Experiment 1. Each point represents the hull pH associated with one bioreactor

**Fig. 6:**
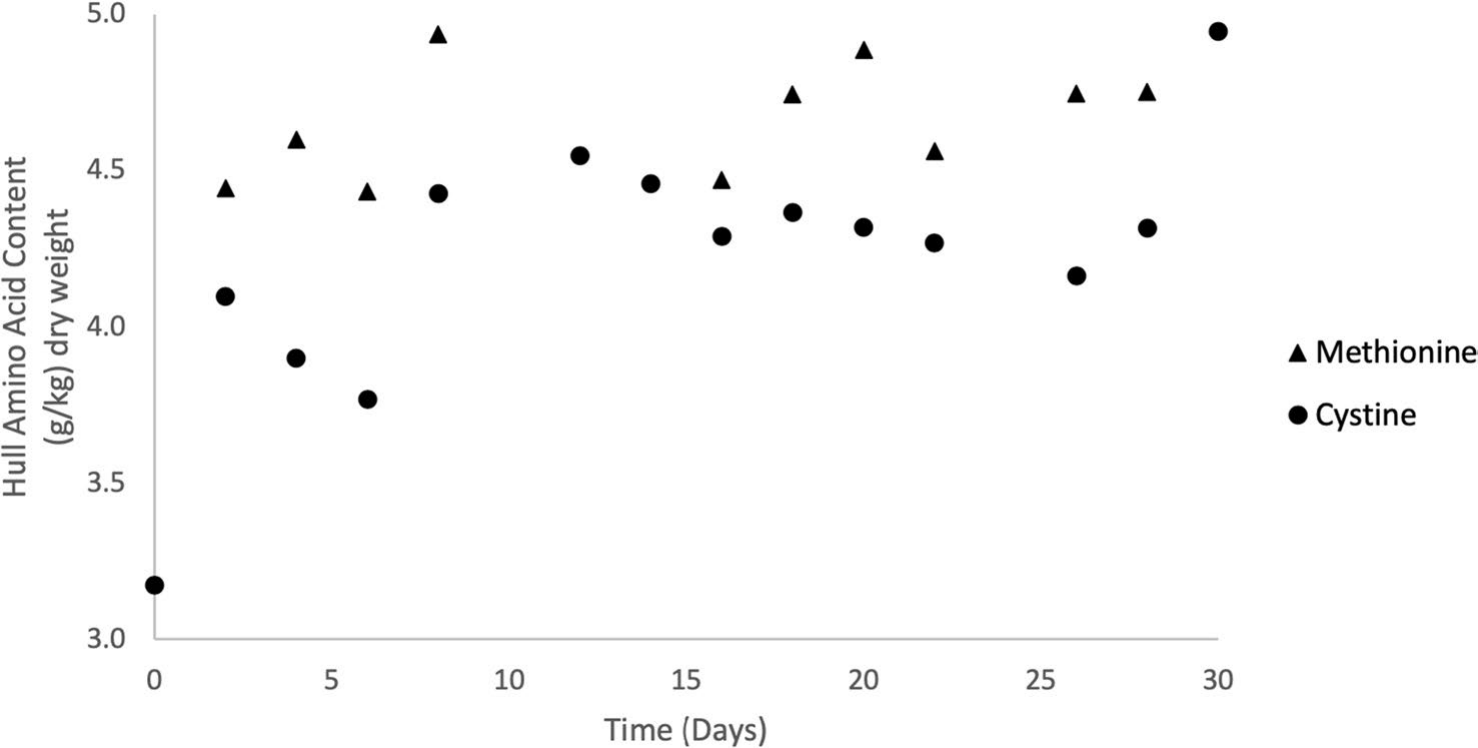
Dynamic changes in methionine and cystine contents of almond hulls during 30-day batch cultivation of BSF larvae in Experiment 1

Hull consumption ranged from 7 to 29% over the first 6 days of the experiment and averaged 24% for the remaining 24 days (Fig. 4). Linear regression of hull consumption data with respect to time for the first 6 days of cultivation showed a significant first-order relationship (Table 3). The pH of hulls increased from 4.72 to 7.92 over the first 8 days and averaged 8.16 for the remaining 22 days (Fig. 5). Hull methionine and cystine contents averaged 4.69 g kg^−1^ dry and 4.22 g kg^−1^ dry, respectively (Fig. 6), throughout the experiment with no significant trends at the beginning of the experiment.

### Semi-batch process

#### Larvae growth

Increasing the number of feedings from one to two over the 14-day experiment significantly impacted the specific larvae growth, average larvae harvest weight and larvae yield (Table 4). Average specific larvae growth was 2.7 times higher when the feedings were increased from one to two, 2.68 g g^−1^ dry and 7.24 g g^−1^ dry, respectively. Average larvae harvest weight was 0.015 g dry larvae^−1^ and 0.033 g dry larvae^−1^ for one and two feedings, respectively, increasing by 2.2 times. Average larvae yield was 4.63 g g^−1^ dry and 14.96 g g^−1^ dry for one and two feedings, respectively, a 3.2 times increase. Varying the C/N ratio for the second feeding did not significantly impact the specific larvae growth, average larvae harvest weight or larvae yield (*P* > 0.05).

**Table 4:** Comparative means of specific larvae growth, harvest average weight, larvae yield, and hull consumption

Number of feedings	C/N ratio of second feeding	Specific larvae growth (g g^−1^ dry)^abc^	Average larvae harvest weight (g dry larvae^−1^)^abc^	Larvae yield (g g^−1^ dry)^abc^	Hull consumption (g g^−1^ dry)^abc^
1	–	2.6 (0.27) B	0.015 (0.001) B	4.63 (1.04) B	0.23 (0.06) B
2	26	7.10 (1.01) A	0.033 (0.003) A	17.02 (5.75) A	0.34 (0.09) AB
2	33	7.06 (0.42) A	0.031 (0.001) A	12.59 (0.83) A	0.42 (0.03) A
2	40	7.46 (0.80) A	0.033 (0.002) A	15.62 (1.13) A	0.36 (0.02) AB
2	47	7.34 (0.72) A	0.034 (0.001) A	14.62 (4.63) A	0.41 (0.09) A

^a^Means and standard deviations in parentheses

^b^Means followed by the same letter within columns are not statistically different at *α* = 0.05 based on Tukey–Kramer HSD test

^c^Three replicates for all treatments

#### Larvae composition

**Fig. 7:**
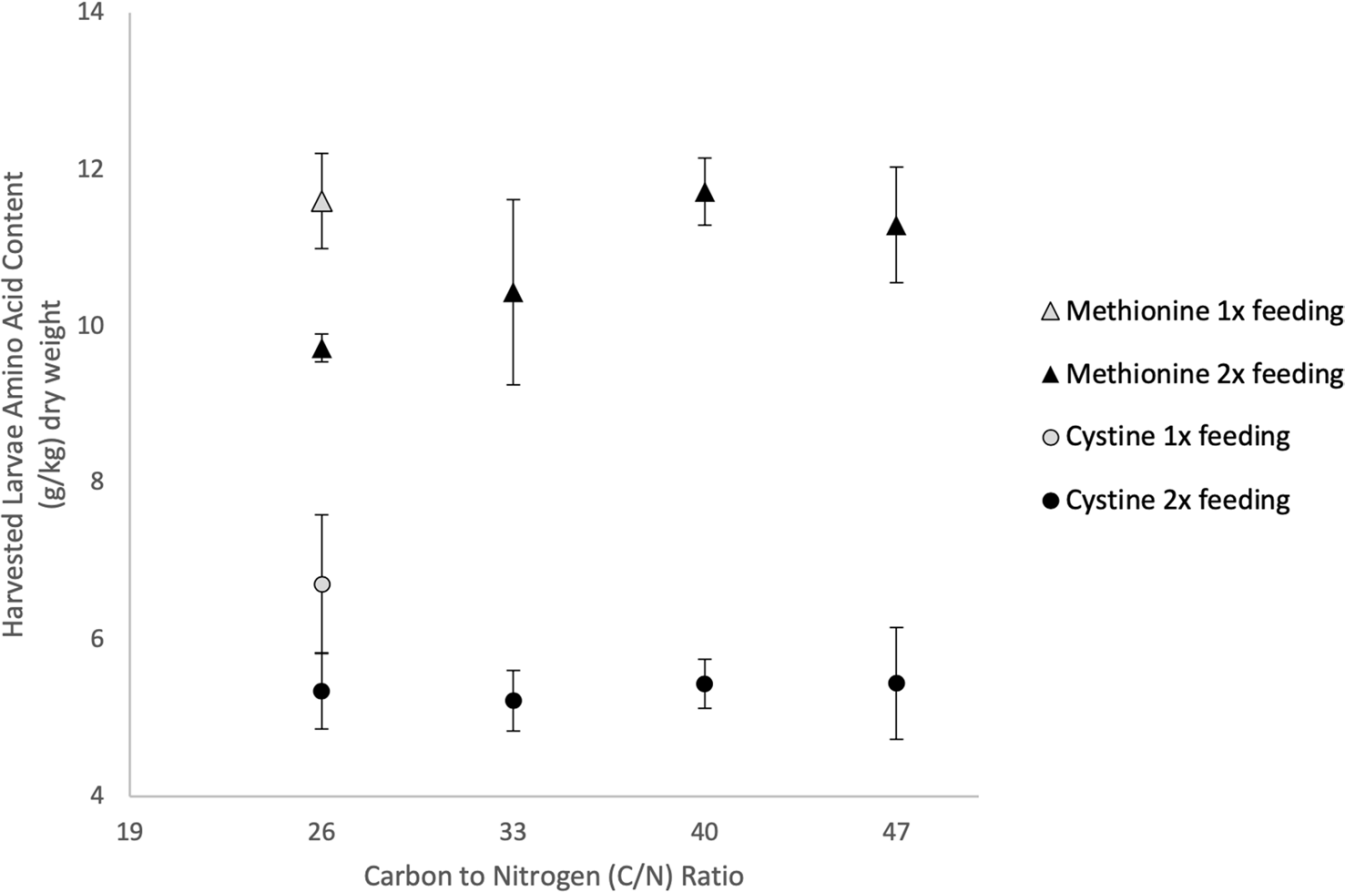
Methionine and cystine contents in black soldier fly larvae grown on almond hulls in Experiment 2. Each point represents the average of bioreactor replicates. Three replicates for all treatments except two replicates for treatments with C/N of 26 and two feedings C/N of 47 due to mishandling of the samples prior to analysis

Average methionine and cystine contents of harvested larvae were 11.6 ± 0.43 g kg^−1^ dry and 6.71 ± 0.63 g kg^−1^ dry, respectively, for the single feeding, and 10.8 ± 0.39 g kg^−1^ dry and 5.36 ± 0.31 g kg^−1^ dry, respectively, for all treatments with two feedings (Fig. 7). Increasing the number of feedings during the cultivation period decreased larvae cystine content nearly 1.3 times, from 6.71 g kg^−1^ dry to 5.36 g kg^−1^ dry. Increasing the number of feedings had a significant and negative first-order effect on larvae cystine content (*P* < 0.05, Table 5), but had no effect on methionine content.

**Table 5:** Statistical analysis of effect of C/N ratio on methionine accumulation and total methionine harvested

Factor	Methionine accumulation^b^ (g kg^−1^)		Total methionine produced^b^ (g dry)		Larvae cystine content^b^ (g kg^−1^ dry)	
	Estimate	*P* value	Estimate	*P* value	Estimate	*P* value
C/N ratio	0.0047	0.0004^a^	0.0007	0.0014^a^	− 0.028	0.21
C/N ratio × C/N ratio	− 0.00014	0.32	− 0.000015	0.62	0.0015	0.65
Number of feedings	− 0.0083	0.005^a^	− 0.0015	0.0058^a^	0.142	0.03^a^

^a^Significant *P* value < 0.05

^b^Three replicates for all treatments except two replicates for treatments with two feedings C/N of 26 and C/N of 47 due to mishandling of the samples prior to analysis

#### Methionine accumulation and production

Regression analysis revealed increasing C/N ratio had significant and positive first-order effects on methionine accumulation and methionine production (*P* < 0.05, Table 5). When the C/N ratio was increased from 26 to 40, the average methionine accumulation increased by 1.2 times, from 0.189 g kg^−1^ dry to 0.233 g kg^−1^ dry. This trend was also observed with total methionine production, which increased nearly 1.3 times (from 30 mg dry to 38 mg dry). Increasing the number of feedings from one to two increased average methionine accumulation by 1.8 times (from 0.104 g kg^−1^ dry to 0.189 g kg^−1^ dry) and increased average methionine production by 1.9 times (from 16 mg dry to 30 mg dry).

#### Hull consumption and pH

Average hull consumption was statistically different and approximately 1.8 times lower for the bioreactors with a single feeding of C/N 26 compared to treatments with two feedings of C/N ratios 33 and 47, ranging from 0.23 to 0.42 g g^−1^ dry, respectively (Table 4). Average pH of spent material varied between 7.86 and 8.55 and was not statistically different between treatments (*P* > 0.05).

## Discussion

### Kinetic growth model

The logistic model successfully predicted BSF larvae accumulation (Table 2). This was consistent upon analysis of data from prior reports (Fig. S2) which showed that BSF larvae follow a logistic curve and reach a stationary growth phase based on the transformation from early prepupa to late pupa (Fig. S2) and prior research [16, 29]. In the model validation, methionine accumulation was overpredicted. This may have been due to several factors. Although the environmental variables were consistent in both experiments using almond hulls as feedstock, the inoculation density, average larvae weight in the inoculum and number of larvae varied between the two experiments (Table 1). In the second experiment (C/N feeding), there were 43% fewer larvae per bioreactor which were double the initial average dry weight at time of inoculation compared to the first experiment (kinetic growth). The larvae in the inoculum of the second experiment were larger than the first experiment, suggesting they were farther along in development and may have reached the stationary phase sooner than larvae in the first experiment. The model does not factor in stage of growth or number of larvae and both were different between experiments which may have contributed to differences in methionine accumulation. A future model might consider stage of growth and number of larvae in the initial conditions.

To further investigate the logistic model and the quality of almond hulls as a feedstock, another data set from prior studies on the dynamic changes of nutrient composition throughout the BSF larvae life cycle was evaluated [29]. In these studies, larvae were cultivated on chicken feed and dry mass of individual BSF larvae was measured. Random sampling was performed at time of feedings on days 4, 6, 7, 9, 12 and 14. Upon examination of these prior data, it can be shown that larvae cultivated on chicken feed also followed a logistic growth curve and the exponential and stationary phases occurred in the BSF larvae life cycle regardless of resources and environment (Fig. S2). However, the kinetic parameters varied with the cultivation feedstock and environmental conditions. BSF larvae grown on chicken feed in a semi-batch operation had approximately 53% higher larvae growth rate (*μ*_max_) compared to BSF larvae grown on almond hulls in batch cultivation, however, the estimated carrying capacity (${X}_{\mathrm{max}}$) was approximately 4–18 times greater for larvae grown on almond hulls, depending on the total amount of chicken feed added throughout the experiment (Table 6).

**Table 6:** Estimated kinetic parameters for black soldier fly larvae

Feedstock	Parameter	Units	Estimate ± SE	References
Almond hulls	${\mu }_{\mathrm{max},b}$	h^−1^	0.019 ± 0.002	Present study
Chicken feed	${\mu }_{\mathrm{max},b}$	h^−1^	0.029 ± 0.0004	[29]
Almond hulls	${X}_{\mathrm{max},b}$^a^	(mg dry larvae^−1^) (g dry hulls)^−1^	0.071 ± 0.003	Present study
Chicken feed	${X}_{\mathrm{max},b}$^b^	(mg dry larvae^−1^) (g dry chicken feed)^−1^	0.004 ± 0.0001–0.021 ± 0.0006	[29]

^a^For the present study using almond hull feedstock, the carrying capacity (${X}_{\mathrm{max}}$) was divided by the average number of larvae per bioreactor

^b^For the study using chicken feed feedstock, the carrying capacity (${X}_{\mathrm{max}}$) was found on an individual larvae basis and divided by the range of dry chicken feed added throughout the experiment [29]

It is well known that cultivation variables such as feedstock, larval density, feeding rate and environmental factors affect the BSF larvae development time and larvae weight. Variation in kinetic parameters using different feedstocks have been shown in prior studies, where BSF larvae had the lowest maximum absolute growth rate and delayed time to reach the maximum absolute growth rate in feedstocks with the lowest protein content [18]. Harnden and Tomberlin [30] reported BSF larvae cultivated on a pork diet took more time to complete larval development than larvae cultivated on beef and grain diets; approximately 23.1% and 139.7% longer, respectively. Barragan-Fonseca et al. [31] investigated the effect of dietary nutrition of feedstock and larval rearing density by mixing chicken feed and cellulose into three different ratios and feeding four different BSF larval densities. In all cases, those fed the diet highest in cellulose and lowest in protein (77% cellulose and 23% chicken feed), had an average 30% longer development time or did not complete larval development at all [31]. Myers et al. [32] reported that increasing the daily feeding rate by 61.4% increased BSF larvae prepupal weight by 34.8% and decreased larval development time by 14.9%. In the present batch cultivation study, BSF larvae were given all feedstock initially, whereas the prior study provided an additional five feedings over the course of 14 days. Increasing the number of feedings using almond hulls as feedstock would likely have a positive effect on the specific larvae growth rate based on results from fed-batch experiments.

Other environmental variables such as type of rearing environment, temperature, and moisture content varied between the present and prior studies and may have contributed to the varying kinetic parameters (Table 7). Temperature has been shown to be a critical parameter for larvae growth. For example, dry weight of larvae was 25% greater for larvae reared on almond hulls at 34 °C compared to 28 °C [15] and 27% greater for larvae reared on pork at 32.2 °C compared to 27.6 °C [30]. In another study, Tomberlin et al. [33] demonstrated temperature differences as little as 3 °C produced significant effects on larval development. In prior experiments using almond hulls as feedstock, average dry weight of larvae was 45% greater when moisture content was increased from 480 g kg^−1^ (wet basis) to 680 g kg^−1^ and a positive linear relationship was observed between moisture content and larvae weight [28]. Almond hulls contain more fibers than chicken feed and can hold more water and this additional water may have increased larvae growth. In other words, the 600 g kg^−1^ wet basis chicken feed may have had more free water available than 690 g kg^−1^ wet basis almond hulls.

**Table 7:** Comparison of cultivation parameters for kinetic experiments

Cultivation variable	Present study	Prior study Liu et al. 2017 [29]
Feedstock	Almond hulls	Chicken feed
Number of feedings during 16-day experimental period	1	16
Total weight of feedstock added throughout experiment (g dry)	200	3200–16,000^a^
Moisture content of feedstock (g kg^−1^ wet basis)	690	600
Aeration type	Forced aeration	Natural convection
Temperature of environment	Incubator setpoint 28 °C	Ambient (unknown)
Cultivation container type	Closed bioreactor	Open basin
Volume of each cultivation container (mL)	1500	11,000
Quantity of larvae per container	143 larvae	220 egg mass

^a^200–1000 dry grams of chicken feed was added daily as needed

In addition to cultivation and environmental variables, the time of harvest can further impact larvae composition including lipid, carbohydrate, amino acid and mineral content. In the present study, methionine content in harvested larvae increased by 54% reaching 17.1 g kg^−1^ at the end of 30 days. In a prior study by Liu et al. [29], the methionine content of black soldier fly increased by 71% after approximately 30 days of growth on chicken feed (Fig. S3). Similar to the present study, linear regression of larvae methionine content revealed significant first-order effects as larvae developed over time with a slope of 0.63 and p value of 0.0003. While the impact of cultivation feedstock on larvae composition is well known, our findings indicate that time of harvest can also be managed to optimize amino acid content such as methionine content.

### Semi-batch cultivation

Several studies have determined the effect of feeding frequency on BSF larvae production. Diener et al. (2009) used chicken feed to cultivate BSF larvae at five daily feeding rates of 12.5, 25, 50, 100 and 200 mg/larvae/day and found that a daily feeding rate of 100 mg/larvae/day was optimal for conversion efficiency and substrate consumption [34]. Another study cultivated BSF larvae on rice straw byproduct at the same daily feeding rates of 12.5, 25, 50, 100 and 200 mg/larvae/day and found the larvae with the highest daily feeding rate of 200 mg/larvae/day yielded the highest prepupal dry weight but the lowest waste reduction efficiency [35]. A study that cultivated BSF larvae on human feces as a sanitation and waste treatment strategy found a single batch feeding resulted in an average of 38% higher prepupal weight compared to six feedings totaling the same amount as the single feeding [36]. In the present study, using the same amount of almond hulls but increasing the feedings from one to two, increased specific larvae growth and yield by approximately three times and average larvae harvest weight by two times. It was observed that the addition of feedstock resulted in an initial increase in temperature within the bioreactors. Prior studies with a similar feedstock demonstrated that increasing temperature from 28 to 34 °C had a significant and positive effect on larvae harvest dry weight, specific larvae growth and larvae yield [15]. For this reason, the increase in temperature associated with addition of feedstock may be one of the contributing factors to the increased growth. Another factor that likely played a role on larvae growth was the time of additional feedings during the logistic growth period. Future experiments could be done to determine optimal feedstock amount, number of feedings and time of feedings for BSF larvae grown on almond hulls.

There are very few studies that have directly investigated the effect of feedstock C/N ratio on the cultivation of larvae. In our previous research, we found that varying C/N ratio using a Pollinator variety of almond hulls as feedstock and urea as a model nitrogen source can affect BSF larvae growth and methionine content. While the direct mechanism of nitrogen amendment on larvae was not investigated in the current study, supplemented nitrogen is important for the growth of microorganisms and synthesis of enzymes necessary to breakdown lignocellulose in nitrogen poor substrates like almond hulls [15]. In the prior study, decreasing C/N ratio from 49 to 16 by increasing amendment of urea decreased larvae methionine by 11% and increased larvae specific growth by approximately 30% [15]. In the present study, a similar relationship was observed with methionine accumulation and total methionine production; where decreasing C/N from 40 to 26 decreased methionine accumulation and total methionine production by 25% and 26%, respectively. Another factor that may have contributed to differences observed in larvae growth is the almond hull type used in the cultivation studies. In another of our previous studies, we compared BSF larvae cultivation on six different almond hull samples. BSF-specific larvae growth (g g^−1^ dry) and harvest average larvae weight (g dry larvae^−1^) were highest for a Monterey variety and approximately 12% and 6.6% higher, respectively, than the average of the Pollinator varieties [37]. The study also reported Monterey hulls had the lowest C/N ratio of 42 compared to the other samples and was approximately 1.7 times lower than the Pollinator varieties, which averaged a C/N of 71 [37]. It is possible that the almond hull nitrogen in the current study using Monterey hulls (C/N of 47) had a higher bioavailability compared to the previous study that used Pollinator hulls. Also, since the C/N ratio was achieved by adding urea to the water in the hull mixture, it is possible nitrogen in urea was more accessible and, therefore, played an important role in the growth and yield of larvae.

## Conclusion

The results demonstrate a logistic model can be used to estimate BSF larvae biomass accumulation when grown on almond hulls and chicken feed. Methionine content of BSF larvae produced on almond hulls showed a linear increasing trend with cultivation time. Increasing the number of feedings from one to two increased the growth, weight and yield of BSF larvae grown on almond hulls. Methionine accumulation and methionine production were significantly impacted by C/N ratio. These results reinforce that careful management of environmental conditions and feedstock need to be considered to optimize larvae production and quality.

## Supplementary Information

Below is the link to the electronic supplementary material.

Supplementary file1 (DOC 244 KB)
