# Supplementary material for: Predicting black soldier fly larvae biomass and methionine accumulation using a kinetic model for batch cultivation and improving system performance using semi-batch cultivation
Source: Bioprocess Biosyst Eng. 2021 Dec 4;45(2):333–44. doi: 10.1007/s00449-021-02663-y (PMC8807430; doi:10.1007/s00449-021-02663-y)
Supplement: Supplementary file 1 — Supplementary file1 (DOC 244 KB) [file 449_2021_2663_MOESM1_ESM.doc]

Table S1: Final pH and moisture content of almond hull feedstock for Experiment 2

| Number of feedings | C/N ratio of second feeding | pH^a^ | Moisture content^a^  (g/kg wet basis) |
| --- | --- | --- | --- |
| 1 | -- | 7.8(0.1) | 734(21) |
| 2 | 26 | 8.5(0.05) | 712(16) |
| 2 | 33 | 8.4(0.09) | 727(5.0) |
| 2 | 40 | 8.4(0.08) | 716(3.4) |
| 2 | 47 | 8.3(0.05) | 724(17) |

^a^ Means and standard deviations in parentheses.


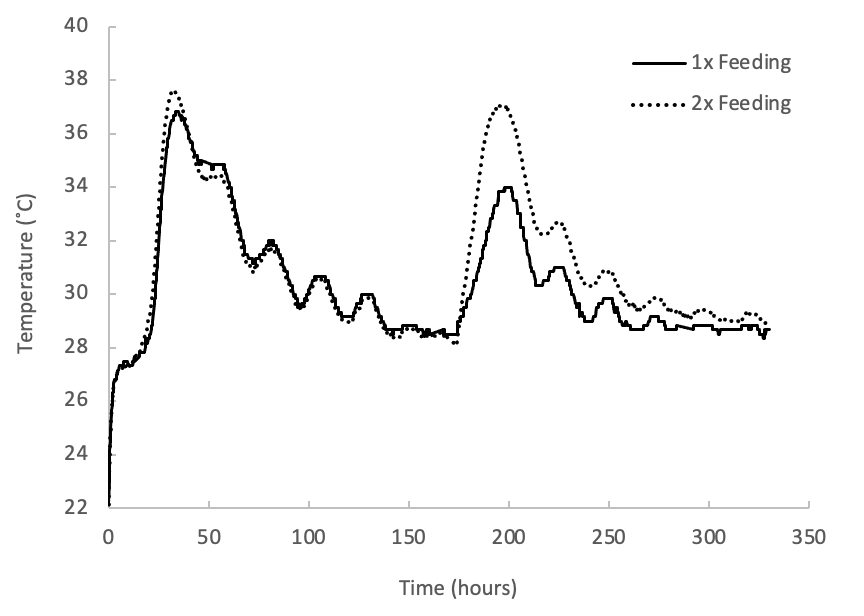


Fig S1: Average temperature data of bioreactors. The solid line represents bioreactors under a batch process and the dotted line represents bioreactors under a semi-batch process. The label (1) indicates the time of the first feeding and the label (2) indicates the time of the second feeding.


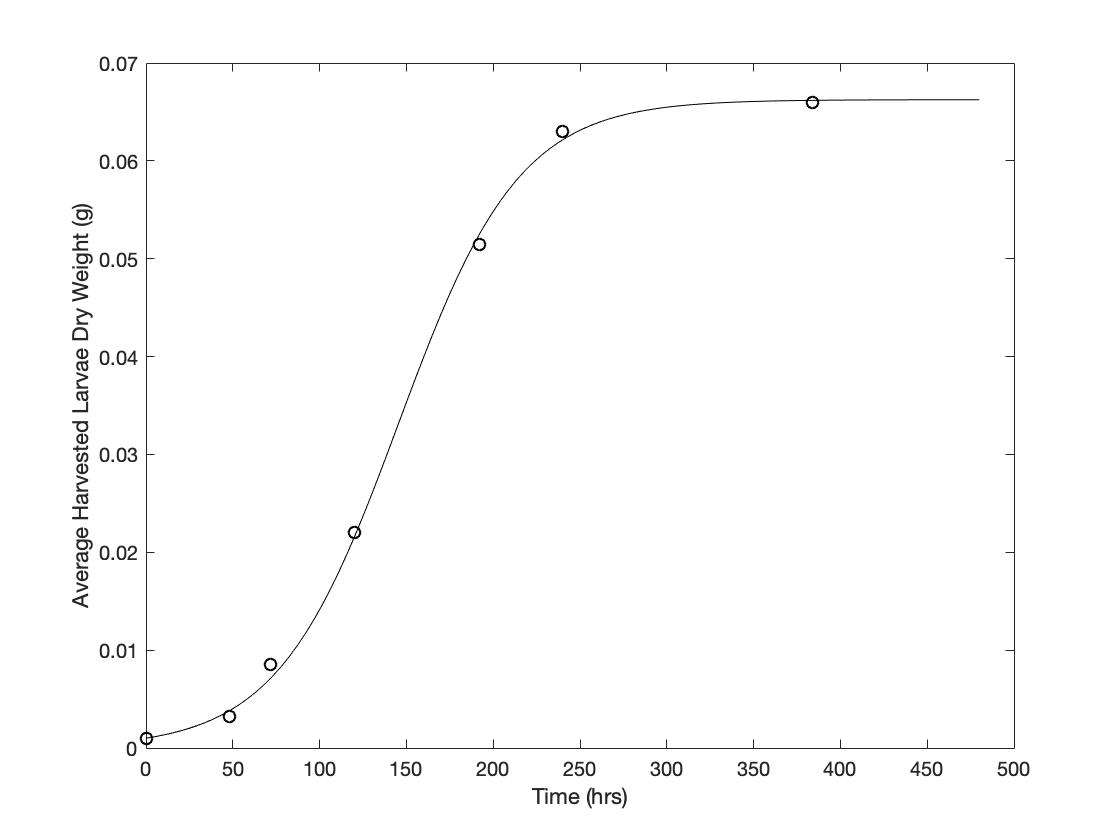


##### Fig. S2: Individual larvae growth data from study by Liu et al. 2017 [37]. The points represent experimental data for larvae reared on chicken feed and the line represents the model fit of the logistic equation.


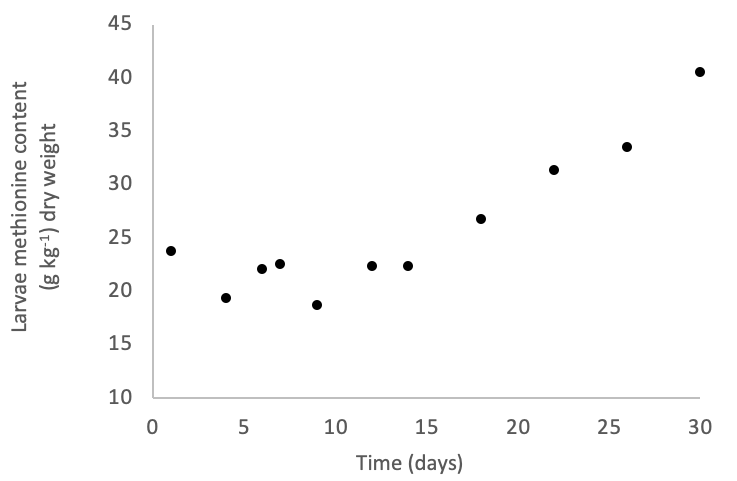


##### Fig. S3: Larvae methionine content data from study by Liu et al. 2017 [37]. The points represent experimental data for larvae reared on chicken feed.
